# VSV infection and LPS treatment alter serum bile acid profiles, bile acid biosynthesis, and bile acid receptors in mice

**DOI:** 10.1128/spectrum.00836-24

**Published:** 2024-09-17

**Authors:** Yamei Li, Yan Luo, Chao Wang, Lei Xu, Xinhua Dai, Yunfei An, Lin He, Dongmei Zeng, Yangjuan Bai, Hua Zhang

**Affiliations:** 1 Department of Laboratory Medicine/Clinical Laboratory Medicine Research Center, West China Hospital, Sichuan University, Chengdu, Sichuan, China; 2 Sichuan Clinical Research Center for Laboratory Medicine, Chengdu, Sichuan, China; 3 Department of Pathology, General Hospital of Western Theater Command, Chengdu, Sichuan, China; 4 Pancreatic Injury and Repair Key Laboratory of Sichuan Province, General Hospital of Western Theater Command, Chengdu, Sichuan, China; Georgia Institute of Technology, Atlanta, Georgia, USA

**Keywords:** VSV infection, LPS treatment, bile acid profiles, bile acid biosynthesis, bile acid receptors

## Abstract

Pathogen infections remain a significant public health problem worldwide. Accumulating evidence regarding the crosstalk between bile acid (BA) metabolism and immune response reveals that BA metabolism regulates host immunity and microbial pathogenesis, making it an attractive target for disease prevention and infection control. However, the effect of infection on circulating BA profiles, the biosynthesis-related enzymes, and their receptors remains to be depicted. Here, we investigated the effect of viral (vesicular stomatitis virus, VSV) and bacterial (lipopolysaccharide, LPS) infections on BA metabolism and signaling. Infection models were successfully established by intraperitoneally injecting VSV and LPS, respectively. VSV and LPS injection significantly changed the circulating BA profiles, with highly increased levels of taurine-conjugated BAs and significant decreases in unconjugated BAs. Consistent with the decreased levels of circulating cholic acid (CA) and chenodeoxycholic acid (CDCA), the expression of BA biosynthesis-related rate-limiting enzymes (*Cyp7a1*, *Cyp27a1*, *Cyp8b1,* and *Hsd3b7*) were significantly reduced. Furthermore, hepatic and pulmonary BA receptors (BARs) expression varied in different infection models. LPS treatment had an extensive impact on tested hepatic and pulmonary BARs, resulting in the upregulation of TGR5, S1PR2, and VDR, while VSV infection only promoted VDR expression. Our study provides insights into the involvement of BA metabolism in the pathophysiology of infection, which may provide potential clues for targeting BA metabolism and BAR signaling to boost innate immunity and control infection.

**IMPORTANCE:**

This study focuses on the crosstalk between bile acid (BA) metabolism and immune response in VSV infection and LPS treatment models and depicts the effect of infection on circulating BA profiles, the biosynthesis-related enzymes, and their receptors. These findings provide insights into the effect of infection on BA metabolism and signaling, adding a more comprehensive understanding to the relationship between infection, BA metabolism and immune responses.

## INTRODUCTION

Infections caused by various pathogens are a significant public health problem affecting tens of thousands of people worldwide. In recent years, intensive evidence has revealed that the mutual regulation of metabolism and the immune response play a crucial role in regulating anti-infection activity, paving the path for exploring therapeutic targets. Bile acids (BAs), as a cluster of downstream metabolites of cholesterol, were initially considered detergents promoting the digestion and absorption of fat and fat-soluble vitamins for many years (1, 2). Now, it has been recognized that BAs are common host and microbial metabolites that regulate host immunity and microbial pathogenesis. Recently, accumulating evidence has revealed that BAs are also important signaling molecules exerting pleiotropic biological functions in controlling energy homeostasis, regulating systemic immunometabolism and inflammation, maintaining immune tolerance, and fighting against infection (2–4), thus making it an attractive target for disease prevention and infection control.

Studies have focused on the relationship between pathogen infection and BA metabolism and signaling in recent years, bringing us more clues and directions to investigate the detailed mechanisms and application prospects. In COVID-19 pneumonia, high levels of serum BAs (≥10.1 µmol/L) are toxic and involved in the inflammatory process and progression to severe and critical clinical stages (5)**,** indicating that circulating BA levels are associated with clinical progression and outcomes of viral infections. Furthermore, a previous study revealed that Hepatitis B virus (HBV) infection enhanced the transcription of BA synthesis and cholesterol uptake-related genes *CYP7A1*, *SREBP2*, *HMGCR* in the liver of both humans and chimeric mice, promoting BA metabolism (6, 7). Conversely, endogenous or exogenous alteration of BA composition or concentration, on the one hand, would influence the HBV gene expression and replication, on the other hand, lower the sensitivity of the host to IFN-α treatment (8, 9). These findings suggest that BA metabolism may play a complicated regulatory effect in viral behaviors and host immunity. Consistently, viral infection would induce the rapid transcription of extrahepatic BA transporters and rate-limiting biosynthesis genes, as well as lead to the intracellular accumulation of BAs in macrophages, which promoted the activation of antiviral signaling via the TGR5-β-arrestin-SRC axis, implying the universal role of BA metabolism in antiviral innate immunity (10, 11). More significantly, intestinal microbiome-derived secondary bile acid (SBA), deoxycholic acid (DCA), has been reported to modulate innate immunity and type I IFN responses to control alphavirus infection (12). And ursodeoxycholic acid (UDCA), a kind of SBAs, has been reported to inhibit the FXR-ACE2 axis and reduce susceptibility to SARS-CoV-2 infection, leading to positive clinical outcomes in SARS-CoV-2 infection (13). These studies highlight the significance of BA metabolism in the pathogenesis and treatment of viral infections.

In addition to acting on viral infection, BA metabolism is closely connected with the intestinal microbiota. The disorder of the intestinal microbiota often leads to changes in BA metabolic pathways. Bacterial infection such as Clostridium *difficile* infection significantly alters the BA profiles, and the SBAs in stool might be potential biomarkers of recurrent Clostridium *difficile* infection (14). Lipopolysaccharide (LPS)-induced endotoxemia significantly changes the composition of circulating BAs in humans (15). More importantly, chenodeoxycholic acid (CDCA), one of the primary BAs (PBAs), can directly inhibit the expression of virulence genes and invasion of epithelial cells in *Salmonella enterica serovar* Typhimurium infection (16). The protective effect of UDCA has also been observed in sepsis-induced lung injury (17). These findings indicated that bacterial infection also works on BA metabolism, and BAs represent a potential target for bacterial infection treatment. In addition, the dysregulation of BA receptors (BARs), such as farnesoid X receptor (FXR), takeda G protein-coupled receptor (TGR5), sphingosine 1-phosphate receptor (S1PR2), and vitamin D receptor (VDR), shows a remarkable effect on host anti-infection activities, but their functions vary in different infection models (4, 13, 18–22). All these findings support that BA metabolism, and its downstream signaling play a pivotal role in all kinds of infections, illustrating the mechanism behind may provide therapeutic opportunities for anti-infection. However, the effect of the infection on circulating BA profiles, the biosynthesis-related enzymes, and its receptors remains to be depicted.

In this study, considering the rapid replication and wide host range of vesicular stomatitis virus (VSV), as well as the fact that LPS is the main structural component on the cell wall outer membrane of all Gram-negative bacteria, which are commonly used in infection model construction, we selected VSV and LPS as agents to construct viral and bacterial infection models, respectively. We characterized the circulating BA profiles, the expression of BA biosynthesis and transport-related enzymes in the liver, as well as the expression of BARs in liver and lung tissues in both models to explore the effect of infection on BA metabolism and signaling, aiming to provide insights into BA metabolism in the pathophysiology of infection.

## MATERIALS AND METHODS

### Animals and infection models

Eight-week-old female C57BL/6J mice were purchased from GemPharmatech (Chengdu, China) and were housed in a pathogen-free animal facility with a 12 h light/dark cycle. For the infection model, age-matched mice were intraperitoneally injected with VSV (1.5 × 10^6^ plaque-forming units per gram body weight, *n* = 14) or LPS (5 mg/kg, *n* = 11) to imitate viral infection and bacterial infection, respectively. For the control group (*n* = 9), age-matched mice were intraperitoneally injected with the same volume of solvent. Twenty-four hours later, mice were euthanized for the collection of serum, lung, and liver samples. All animal experiments were performed in accordance with the National Institute of Health Guide for the Care and Use of Laboratory Animals and approved by the ethics committee of the General Hospital of Western Theater Command, Chengdu, China.

### RNA extraction and quantification by quantitative real-time PCR

Total RNA was obtained from liver and lung tissues using Trizol reagent (Invitrogen, USA) and reverse-transcribed using PrimeScript RT reagent Kit with gDNA Eraser (Takara, Japan). Quantitative real-time PCR analysis was performed on C1000 Thermal Cycler (BIO-RAD, USA) with TB Green Premix Ex Taq II (Tli RNaseH Plus) (Takara, Japan) according to the manufacturer’s instructions. The 2^−∆∆Ct^ method was used to determine the relative expression levels of target genes, and *Gapdh* served as an internal mRNA quantity control. The primer sequences of the targeted genes were listed in Table S1.

### Western blotting

Total protein was extracted from the liver using RIPA lysis and extraction buffer (Servicebio, China) supplemented with protease inhibitor cocktail (Cell Signaling Technology, USA). The protein concentration was determined with the BCA assay (Servicebio, China). An equal amount of protein was loaded on a 4%–20% gradient gel and transferred onto PVDF membranes. Blots were blocked with 5% skimmed milk powder in TBST for 1 h and incubated with primary antibodies at 4**°**C overnight. Then, after incubation with secondary antibodies for 1 h at room temperature, SuperSignal ECL reagent (Thermo Scientific, USA) was used for image development. The diluted antibodies used in this study were listed as follows: anti-GAPDH (GB15004, 1:1,000, Servicebio, China), anti-RIG-I (3743, 1:1,000, Cell Signaling Technology, USA), anti-p-TBK1(5483, 1:1,000, Cell Signaling Technology, USA), anti-p-p65 (3033, 1:1,000, Cell Signaling Technology, USA), anti-p-STAT1 (9167, 1:1,000, Cell Signaling Technology, USA), anti-CYP7A1 (ab65596, 1:1,000, abcam, UK), anti-CH25H (sc-293256, 1:1,000, Santa Cruz Biotechnology, USA), anti-SHP (sc-271511, 1:1,000, Santa Cruz Biotechnology, USA), anti-mouse IgG, HRP-linked Antibody (7076, 1:2,000, Cell Signaling Technology, USA), anti-rabbit IgG, HRP-linked Antibody (7074, 1:2,000, Cell Signaling Technology, USA). The protein bands were semi-quantified with Image J software.

### High-performance liquid chromatography-MS/MS

Serum BA profiles were measured by using a Waters ACQUITY UPLC system coupled with a Waters XEVO TQ-S mass spectrometer with an ESI source controlled by Masslynx 4.1 software. BA assay kit was purchased from Qlife Lab (Nanjing, China). Detailed procedures for the determination of BA metabolites are the same as our previously published protocol (23). Fifteen BAs including cholic acid (CA), CDCA, glycocholic acid (GCA), taurocholic acid (TCA), glycochenodeoxycholic acid (GCDCA), taurochenoxycholic acid (TCDCA), DCA, lithocholic acid (LCA), UDCA, glycodeoxycholic acid (GDCA), glycolithocholic acid (GLCA), aurodeoxycholic acid (TDCA), tauroursodeoxycholic acid (TUDCA), glycoursodeoxycholic acid (GUDCA), and taurolithocholic acid (TLCA) were detected in this study, but the levels of 5 BA metabolites (GCDCA, GDCA, GCLA, GUDCA, and TLCA) were extremely low in the mice serum. Therefore, we only focused on the 10 detectable BAs in the following analyses.

### Enzyme-linked immunosorbent assay

Serum concentration of taurine was quantified with commercially available enzyme-linked immunosorbent assay kit (ELK Biotechonology, Wuhan, China), strictly according to the manufacturer’s instructions. Competitive inhibition enzyme immunoassay technique was employed in this assay.

### Histopathological assessment and immunohistochemistry staining

For morphological examination, the lung and liver tissues from each mouse were rapidly removed and fixed in freshly prepared 4% neutral polyformaldehyde. After dehydration, the lung and liver specimens were embedded in paraffin, cut 5 µm in thickness, and stained with hematoxylin and eosin (H&E). For immunohistochemistry (IHC) staining, specimens were cut 4 µm in thickness, and immunohistochemical staining of BARs was performed by the streptavidin–biotin system. Deparaffinized specimens were incubated with fresh 0.3% hydrogen peroxide and then incubated with anti-FXR (25055-1-AP, 1:300, Proteintech, China), anti-TGR5 (ab72608, 1:300, abcam, UK), anti-S1PR2 (21180-1-AP, 1:300, Proteintech, China), and anti-VDR (67192-1-Ig, 1:300, Proteintech, China) for 1 h at room temperature. Finally, the specimens were processed routinely with streptavidin-biotin and followed by counterstaining with H&E. All sections were investigated by a light microscope. IHC plots were semi-quantified with Image J software.

### Statistical analyses

Data were presented as mean ± standard deviation. Statistical tests were performed using Student’s *t*-test, one-way ANOVA, and the generalized Wilcoxon test. Due to the abnormal distributions of the data sets, spearman correlation analyses were performed to explore the correlations between serum taurine level and individual taurine-conjugated BAs. All statistical analyses and graphs were done with GraphPad Prism (Version 9.0.1, La Jolla, CA, USA). Principle component analysis was completed with MetaboAnalyst (Version 6.0, https://www.metaboanalyst.ca). A difference of *P* < 0.05 was considered significant.

## RESULTS

### Infection models were successfully established

**Fig 1:**
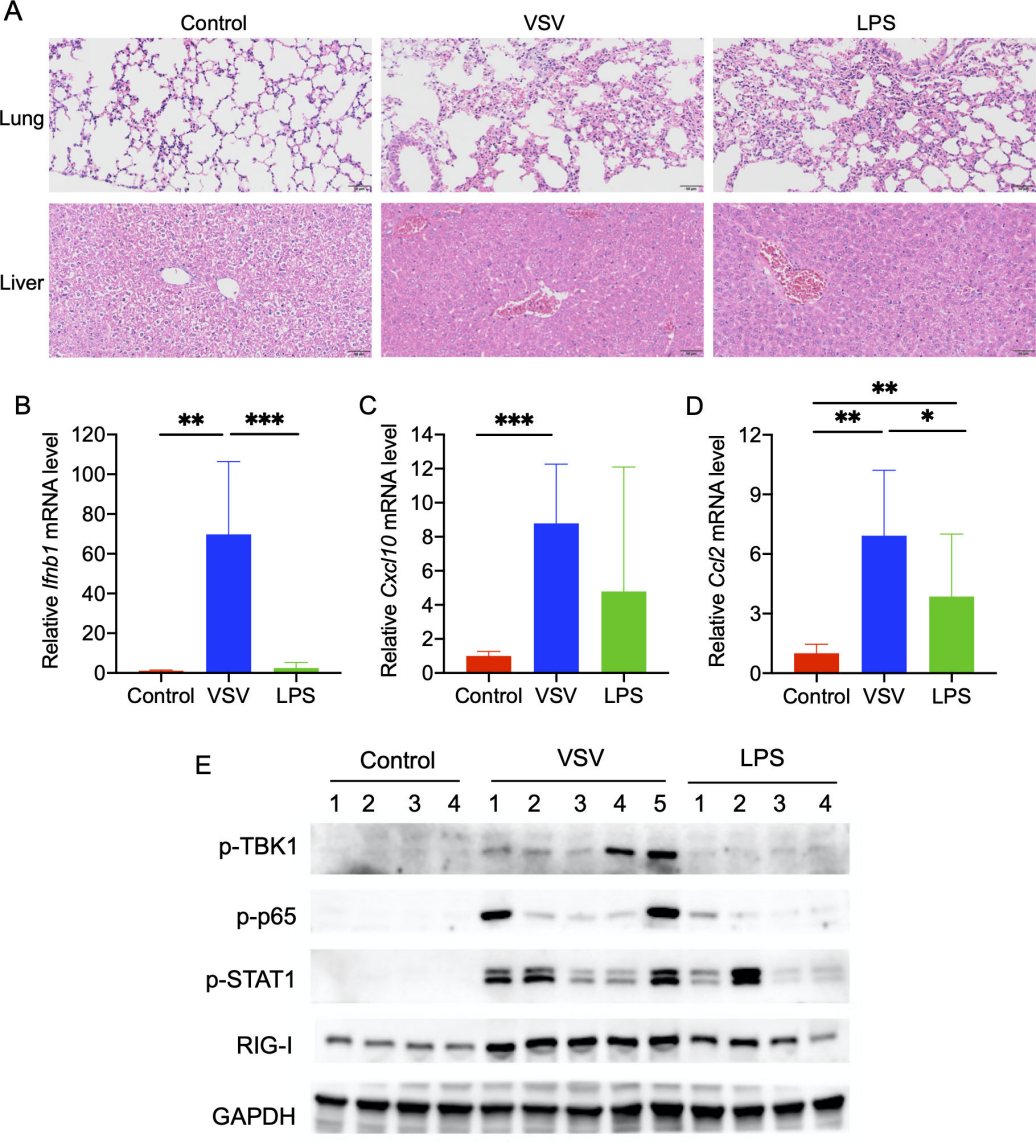
Infection models were successfully established by intraperitoneal injection of VSV (1.5 × 10^6^ plaque-forming units per gram body weight) and LPS (5 mg/kg). (**A**) H&E staining of lung and liver tissues. Scale bar, 50 µm. (**B–D**). Relative mRNA levels of *Ifnb1* (**B**), *Cxcl10* (**C**), and *Ccl2* (**D**) in liver tissues. (E) Immunoblotting analysis of p-TBK1, p-p65, p-STAT1, and RIG-I in liver tissues.

Viral and bacterial infection models were established by intraperitoneal injection of VSV and LPS, respectively. To assess whether the models were successfully established, we evaluated the H&E staining of lung and liver tissues, quantified the transcriptional expression of inflammatory molecules, and determined the activation of innate immune signaling. Histopathology results exhibited obvious focal hemorrhage, inflammatory cell infiltration, and diffuse alveolar collapse with increased thickness of alveolar walls in lung tissues after VSV and LPS treatment. Livers from the VSV and LPS-treated groups also showed massive cytoplasmic exudation, necrosis, and congestion of the central vein (Fig. 1A). VSV and LPS treatment induced the transcription of the hepatic classic antiviral gene *Ifnb1* and inflammatory genes *Cxcl10* and *Ccl2* (Fig. 1B through D). The activation of innate immune signaling was observed, as evidenced by elevated levels of p-TBK1, p-p65, p-STAT1, and RIG-1 in livers from the VSV and LPS-treated groups (Fig. 1E). All these data supported the successful establishment of the infection models.

### VSV and LPS treatment significantly altered the serum BA profiles

**Fig 2:**
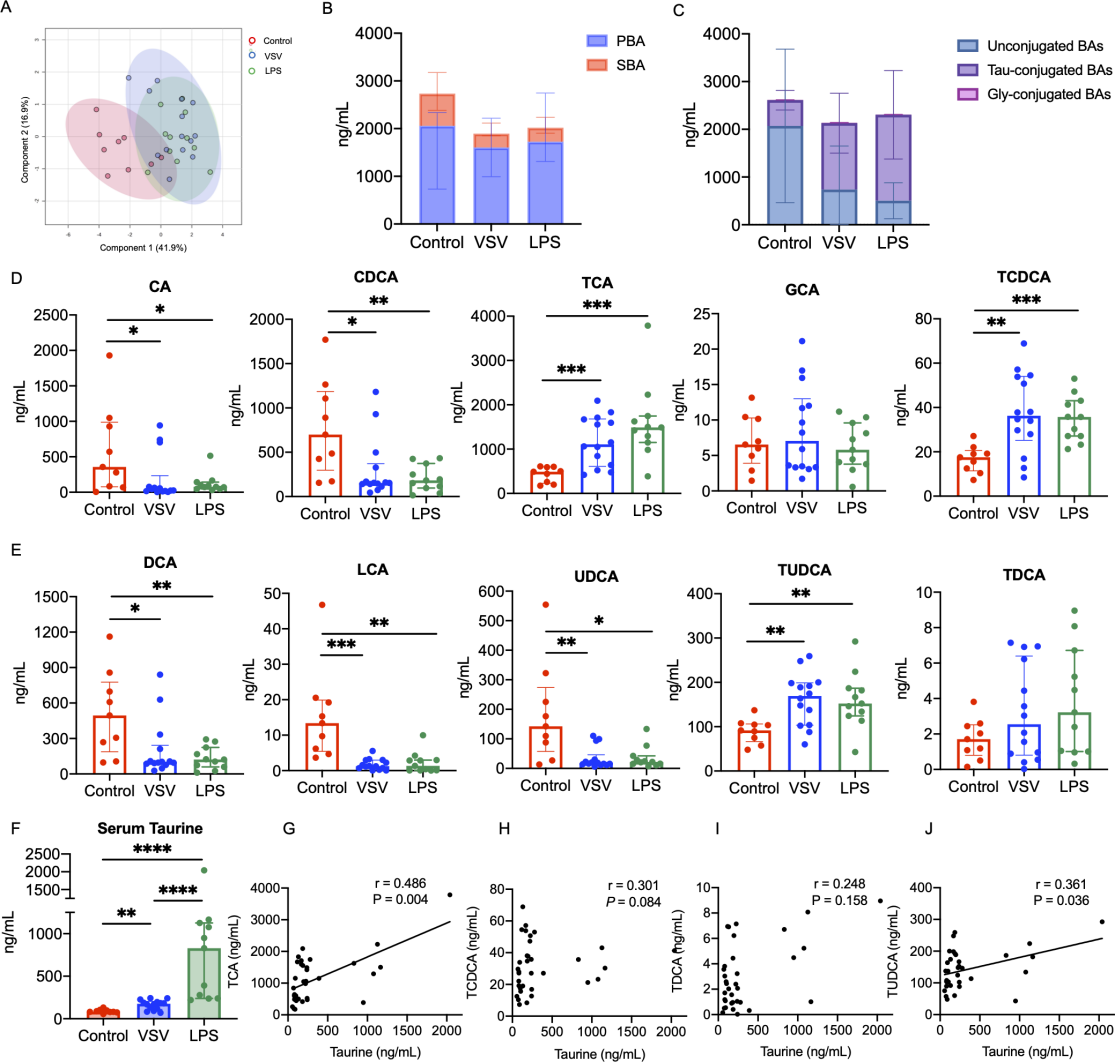
Comparison of serum BA profiles in control, VSV, and LPS groups. (A) PLS-DA score plot for comparison of the serum BA profiles among three groups. 95% confidence regions were circled as ellipses. (B) Serum concentrations of PBAs and SBAs. (C) Serum concentrations of unconjugated and taurine/glycine-conjugated BAs. (D) Individual concentration of 5 PBAs (CA, CDCA, TCA, GCA, and TCDCA). (E) Individual concentration of 5 SBAs (DCA, LCA, UDCA, TUDCA, and TDCA). (F) Concentration of serum taurine in control, VSV, and LPS groups. (G–J) Spearman correlations between serum taurine concentration and TCA (G), TCDCA (H), TDCA (I), TUDCA (J) levels. Note: PBAs, primary BAs, the sum of CA, CDCA, TCA, GCA, and TCDCA; SBAs, secondary BAs, the sum of DCA, LCA, UDCA, TUDCA, and TDCA; *P < 0.05; **P < 0.01; ***P < 0.001.

A total of 15 BA metabolites were quantified using the high-performance liquid chromatography-MS/MS. However, considering extremely low levels of GCDCA, GDCA, GCLA, GUDCA, and TLCA in mouse serum, we only statistically analyzed the rest of the 10 BA metabolites. The supervised partial least squares-discriminant analysis (PLS-DA) plot showed that the BA profiles can significantly separate the infection models from control mice but cannot be separated from each other in the two experimental groups (Fig. 2A). Compared with control mice, the concentrations of total taurine-conjugated BAs were significantly increased, while unconjugated BAs and SBAs (the sum of DCA, LCA, UDCA, TUDCA, and TDCA) were significantly decreased in the serum of both VSV and LPS treated mice. No significant differences were observed between the two experimental groups in the levels of PBAs (the sum of CA, CDCA, TCA, GCA, and TCDCA), SBAs, unconjugated and conjugated BAs (Fig. 2B and C). Further quantification of individual BAs demonstrated that CA, CDCA, TCA, DCA, UDCA, and TUDCA accounted for most of the detected BAs. The comparison results showed that CA, CDCA, DCA, LCA, and UDCA were significantly decreased, while TCA, TCDCA and TUDCA were dramatically elevated in VSV and LPS groups (Fig. 2D and E). In addition, VSV and LPS treatment significantly increased the serum level of taurine, with the LPS group showing the highest level (Fig. 2F). Correlation curves displayed that TCA and TUDCA concentrations positively correlated to serum taurine level (Fig. 2G and J).

### Effects of VSV and LPS treatment on the expression of hepatic BA synthesis and transport-related enzymes

**Fig 3:**
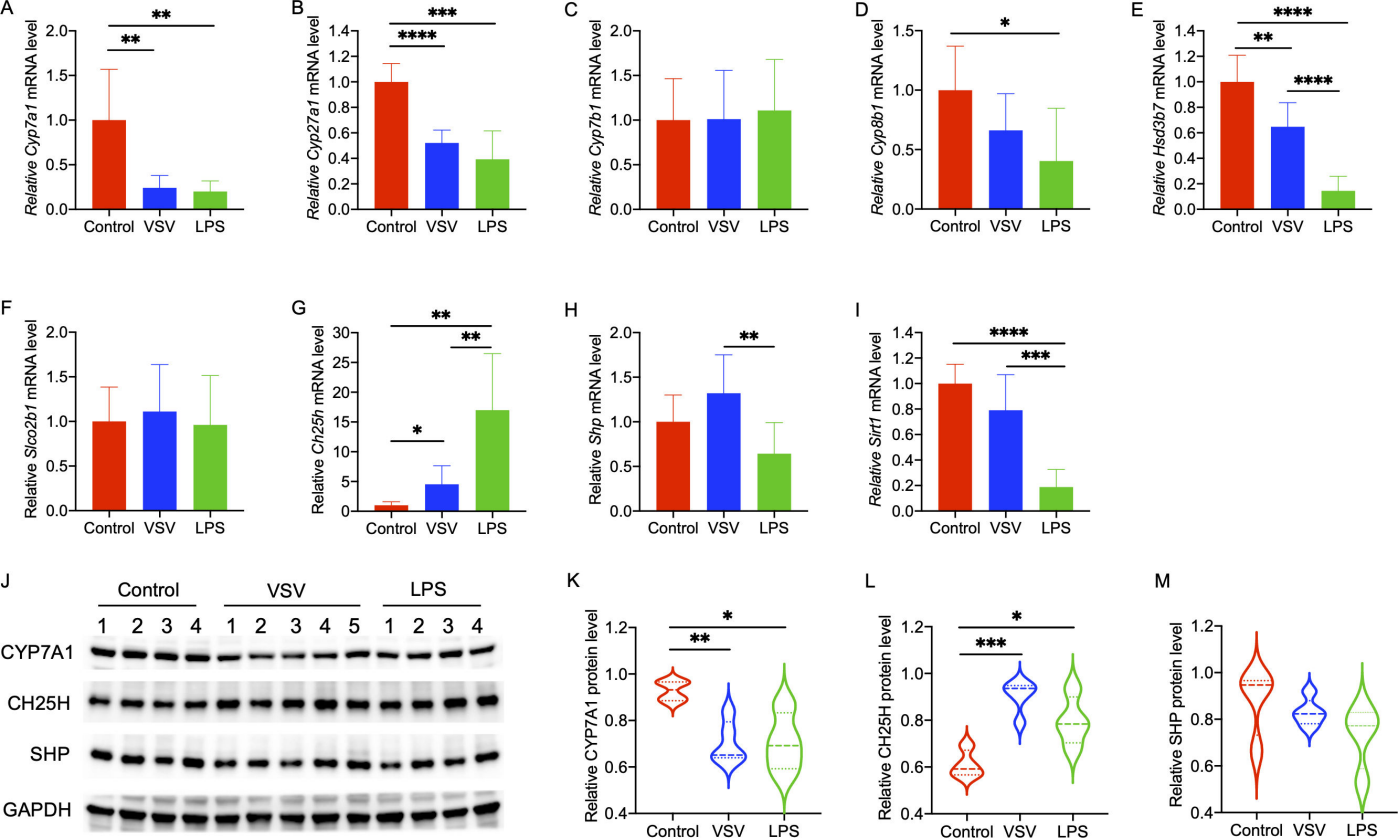
The relative mRNA and protein levels of BA synthesis and transport-related enzymes in the liver of control, VSV, and LPS groups. (A–I) Relative mRNA levels of *Cyp7a1*(A)*, Cyp27a1*(B)*, Cyp7b1*(C)*, Cyp8b1* (D)*, Hsd3b7* (E)*, Slco2b1* (F)*, Ch25h* (G)*, Shp* (H), *and Sirt1*(I). (J) Western blotting analysis of CYP7A1, CH25H, and SHP protein expression among groups. (K–M) Relative protein expression of CYP7A1 (K), CH25H (L), and SHP (M).

As the liver is one of the main organs responsible for BA synthesis and metabolism, we further determined the impact of infections on the expression of BA synthesis and transport-related genes and proteins in the liver. We observed significant transcriptional impairment of the BA rate-limiting biosynthesis enzyme and transporter genes (*Cyp7a1*, *Cyp27a1*, *Cyp8b1*, *Sirt1,* and *Hsd3b7*), while the *Ch25h* (an enzyme converts cholesterol to 25-hydroxycholesterol) mRNA level was significantly induced in both VSV and LPS groups. Both VSV and LPS had no marked effects on the transcription of hepatic *Cyp7b1* and *Slco2b1* (Fig. 3A through I). The protein levels of CYP7A1, CH25H, and SHP showed the same alteration trends as their corresponding mRNA levels (Fig. 3J through M), confirming that VSV and LPS treatment would significantly affect the expression of some BA synthesis-related enzymes.

### Effects of VSV and LPS treatment on the expression of hepatic and pulmonary BARs

**Fig 4:**
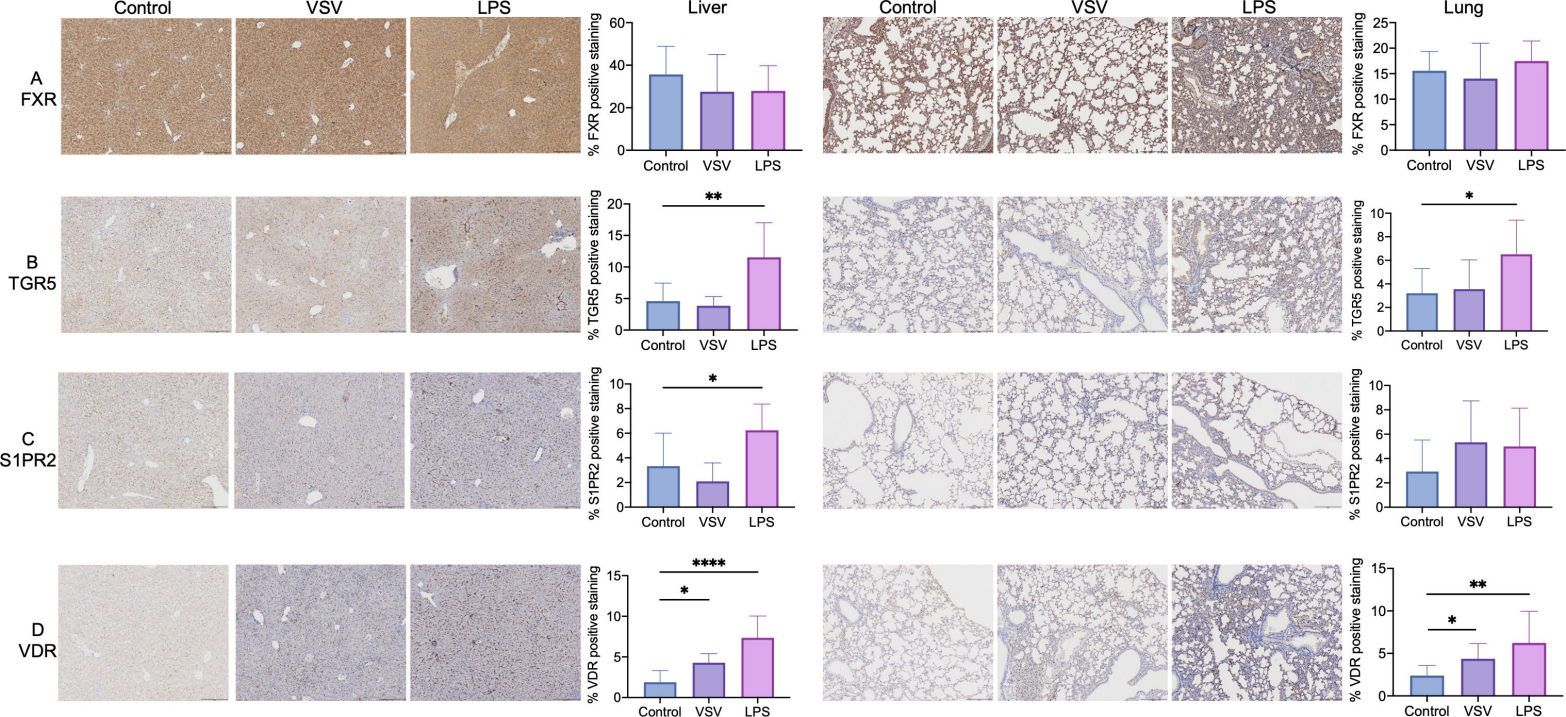
IHC staining of BARs in the liver (left panel) and lung (right panel) tissues of control, VSV, and LPS groups. (**A**) IHC staining and semi-quantification of FXR. (**B**) IHC staining and semi-quantification of TGR5. (**C**) IHC staining and semi-quantification of S1PR2. (**D**) IHC staining and semi-quantification of VDR. Scale bar, 200 µm.

As BAs are pivotal signaling molecules involving a series of biological functions through their interaction with BARs, we conducted the IHC staining of BA-activated nuclear receptors (FXR and VDR) and membrane G protein-coupled bile acid receptors (GPCRs, TGR5, and S1PR2) in the liver and lung to figure out the impact of VSV and LPS on the expression of BARs. As shown in Fig. 4, FXR ubiquitously and highly expressed in hepatocytes and alveolar cells, but there is no significant difference among the three groups. When mice were treated with LPS, the expression of hepatic TGR5 and S1PR2, and pulmonary TGR5 were significantly increased compared to the control group, while VSV has no impact on TGR5 and S1PR2 expression in both tissues. Both VSV- and LPS-treated mice demonstrated obviously higher VDR expression in the liver and lung tissues than that in control mice.

## DISCUSSION

In the present study, we investigated the effect of two infection models (VSV and LPS stimulation) on BA metabolism and signaling. We found that both VSV infection and LPS administration significantly changed the circulating BA profiles, with highly increased levels of taurine-conjugated BAs and significantly decreased levels of unconjugated BAs. The expression of BA biosynthesis-related rate-limiting enzymes (*Cyp7a1*, *Cyp27a1*, *Cyp8b1,* and *Hsd3b7*) was significantly reduced, which was consistent with the decreasing level of circulating CA and CDCA. The expression of hepatic and pulmonary BARs varied upon different infection models. LPS treatment possessed an extensive impact on tested hepatic and pulmonary BARs, resulting in the upregulation of TGR5, S1PR2, and VDR, while VSV infection showed a limited influence on tested BARs, which only promoted VDR expression. Our study provides insight into the effect of infection on BA metabolism and possible clues for targeting BA metabolism and BARs signaling to boost innate immunity and control infections.

PBAs are synthesized in the liver through two pathways known as the classical and alternative biosynthetic pathways. In the classical pathway, cholesterol was converted to 7α-hydroxycholesterol by the first rate-limiting enzyme, Cyp7a1, and then the intermediate was converted to 7α-hydroxy-4-cholesten-3-one by Hsd3b7, which was required for the generation of CA and CDCA. In the alternative pathway, Cyp27a1 was responsible for the transformation of cholesterol to 27-hydroxycholesterol and then converted to CDCA by Cyp7b1 (24). In the current study, we observed that under the challenge of VSV and LPS, the expression of hepatic rate-limiting enzymes Cyp7a1, Cyp27a1, Cyp8b1, and Hsd3b7, as well as circulating CA and CDCA were all significantly decreased in mice, suggesting that infections may interfere with the synthesis process of BAs in the liver. This was partially consistent with Inoue T’s results, in which chronic hepatitis C alters the intestinal BA profiles and results in imbalance of BA biosynthesis in patients (25). However, these results were different from Hu MM’s study which demonstrated that both DNA virus (HSV-1) and RNA virus (SeV) induced the transcription of BA biosynthesis-related enzymes (CYP7A1, CYP7B1, CYP27A1, and transporter SLCO1B2) in human monocytic THP1 cells (10). Oehler et al. also found that HBV infection would upregulate hepatic CYP7A1 expression in human liver chimeric mice (6). These data indicated that BA metabolism is a targeting process of pathogen infections, but this process varies in pathogens, host species, and cell types. Thus, it is necessary to comprehensively investigate the detailed changes and related mechanisms of BA metabolic pathways under specific conditions.

In addition, we observed hepatic CH25H was elevated at both mRNA and protein levels when facing VSV and LPS, which was consistent with the previously reported data (26). CH25H, a hepatic enzyme catalyzing the hydroxylation of cholesterol to 25-hydroxycholesterol(25HC), has been proved to be an antiviral interferon-stimulated gene (ISG), which protected mice from viral infection by producing 25HC to broadly inhibit viral entry (26). This process confers the antiviral state of IFNs and also indicates that CH25H participates in the antiviral process of our VSV infection group. In a diet-induced steatosis model, CH25H has been reported to positively regulate CYP7A1 and CYP27A1 and reduce small heterodimer partner (SHP), thus increasing the synthesis and excretion of BAs, drastically reducing the high-fat diet-induced hepatic steatosis (27). This expression pattern was different from the discovery in our infection models. The upregulation of CH25H and downregulation of CYP7A1 in the current study suggest that pathogen infection may not only affect the BA metabolism, but also cholesterol metabolism, which needs to be further confirmed. What’s more, these findings indicate CH25H as an interesting point regarding cholesterol metabolism and BA metabolism in infectious diseases.

After synthesis from classic and alternative pathways, the products then conjugated with glycine or taurine to increase the water solubility of BAs, promoting their secretion into the bile. We observed that taurine-conjugated BAs account for most of the conjugated BAs in mice, while glycine-conjugated BAs were barely detected, which was opposite to that in humans (28). In addition, the serum level of taurine was significantly increased in VSV and LPS treatment models. Under the condition of infection, the circulating taurine-conjugated BAs (i.e., bile salts) were increased, and the unconjugated SBAs were dramatically decreased, which was consistent with the elevation of taurine-conjugated BAs in the cecal contents of the attenuated strain of the food-borne pathogen *Yersinia pseudotuberculosis* infected mice (29). The exact mechanisms are unclear so far. It has been reported that infection alters the composition and decreases the abundance of various bile salt hydrolases (BSH)-producing bacteria and then reduces the level of microbiota-derived BSHs, which act as the gateway enzyme, involving in the deconjugation of the tauroconjugated or glycoconjugated PBAs to generate their unconjugated counterparts (30). In our study, we speculated that VSV infection or LPS treatment may weaken the deconjugation process via reducing the microbiota-derived BSHs. Also, there is a possibility that the process of BAs amidating with taurine might be enhanced in the liver, which cannot be ruled out. As for the biological role of taurine-conjugated BAs in infection, Apollo Stacy et al. revealed that the BA-derived taurine acted as a nutrient to train the microbiota, which promote its resistance to subsequent infection (29). Exogenous supplementation of taurine alleviated multi-organ injuries by reducing inflammatory cell infiltration and TNF-α expression in septic mice (31). All these findings indicated that the higher level of taurine and taurine-conjugated BAs may contribute to the control of infection, which requires further experiments to confirm. Notably, besides taurine and glycine, other amino acids including phenylalanine, tyrosine, and leucine were also reported to conjugate BAs that were regarded as novel BAs. This process is largely associated with the microbiome activities (32). Moreover, vastly underappreciated diversity and modification of BAs, such as polyamine-conjugated BAs, were discovered recently using untargeted LC-MS/MS, suggesting that it is far more complex than we previously recognized in BA metabolism (33, 34). Further revealing the interaction between those novel BAs and various infections would be of great significance to comprehensively understand the pathophysiology of BAs in the field of infection.

It is worth noting that the function of BAs mostly depends on the signaling triggered by their receptors, which includes nuclear receptors (such as FXR and VDR) and G-protein coupled receptors (GPCRs, such as TGR5 and S1PR2). Previous studies revealed that BARs participate in regulating innate immunity (4, 20–22, 35). We, therefore, explored the effect of infection on the expression of these BARs in liver and lung tissues. Our data showed that LPS stimulation upregulated TGR5, S1PR2, and VDR expression in both liver and lung tissues, while VSV treatment only promoted VDR expression in both tissues. Otherwise, FXR expression remained unchanged among the infection models in liver and lung tissues. As the most affected BARs in our infection models, VDR has been reported to involve in *Helicobacter pylori* and *Salmonella Typhimurium* infection, promoting innate immunity against bacterial infection (20, 36). Considering its upregulated expression upon VSV and LPS treatment, VDR may participate in the activation of innate immunity and promote immune defense against pathogens. But the detailed mechanisms involving VDR expression and activation of innate immunity remain to be further determined. TGR5 and S1PR2 are another two BARs, whose expression has been significantly upregulated upon LPS stimulation in both liver and lung tissues. It is known that TGR5 is the most extensively studied BAR, and numerous studies have investigated its role in infection, inflammasome activation, and innate immunity (4, 37). A recent study showed that TGR5 plays an important role in antiviral innate immunity, involving BAs accumulation via TGR5-β-arrestin-SRC axis, AKT/IRF3-signaling (10, 19, 38). Interestingly, TGR5 is also an ISG and viral infection upregulates TGR5 expression in macrophages (19), which was consistent with our discovery in the LPS treatment model. As for S1PR2, it has been reported to suppress phagocytosis, promote macrophage pyroptosis, and impair antimicrobial defense in the pathogenesis of sepsis (21, 22). These observations indicate that the expression level of TGR5 and S1PR2 is closely related to the pathogenesis of infection in liver and lung tissues. However, more indicators of infection should be dynamically monitored and carefully investigated to fully understand their role in infection.

There are several limitations in this study. First, due to the limited spectrum of detectable BA species in the BA assay kit, we did not detect the level of muricholic acids (MCAs) that are specific to mice. It prevented the comprehensive analysis of BA profiles alteration in mice and affected the completeness of our results. Second, only VSV and LPS were used to establish the infection models in mice, the effect of other pathogens such as DNA virus and gram-positive bacteria on BAs metabolism remains to be investigated. Third, although we depicted the characteristics of circulating BA profiles, hepatic BA synthesis-related enzymes and BARs under the condition of infection, the lack of comprehensive information makes it difficult to figure out the deep associations between these alterations. It is necessary to analyze the potential detailed mechanisms to combine these fragmented results into a whole.

In conclusion, BA metabolism is complex as it involves the host, microbiota, and multiple organs. Deciphering the roles of BAs is complicated but important to fight against infection. In this study, we identified that infection inhibited the transcription of some key rate-limiting enzymes of BAs in the liver, the composition of circulating BAs was significantly altered with a lower level of unconjugated BAs and a higher level of taurine-conjugated BAs in infection groups. In addition, TGR5, S1PR2, and VDR were the main BARs that have been affected by the infection. While our findings are correlative, they provide theoretical information for the deep investigation of the detailed association and mechanism between BA metabolism and pathogen infection.

## SUPPLEMENTAL MATERIAL

Table S1 spectrum.00836-24-s0001.docx The primer sequences of the targeted genes.

ASM does not own the copyrights to Supplemental Material that may be linked to, or accessed through, an article. The authors have granted ASM a non-exclusive, world-wide license to publish the Supplemental Material files. Please contact the corresponding author directly for reuse.
