# Supplementary material for: VSV infection and LPS treatment alter serum bile acid profiles, bile acid biosynthesis, and bile acid receptors in mice
Source: Microbiol Spectr. 2024 Sep 17;12(11):e00836-24. doi: 10.1128/spectrum.00836-24 (PMC11537081; doi:10.1128/spectrum.00836-24)
Supplement: Table S1 — The primer sequences of the targeted genes. [file spectrum.00836-24-s0001.docx]

| **Number** | **Gene** | **Primer Forward (5’ to 3’)** | **Primer Reverse (3’ to 5’)** |
| --- | --- | --- | --- |
| 1 | mm-Ifnb1 | ATG AGT GGT GGT TGC AGG C | TGA CCT TTC AAA TGC AGT AGA TTC A |
| 2 | mm-Cxcl10 | AGT GCT GCC GTC ATT TTC TGC | CAA GCT TCC CTA TGG CCC TCA |
| 3 | mm-Ccl2 | TTA AAA ACC TGG ATC GGA ACC AA | GCA TTA GCT TCA GAT TTA CGG GT |
| 4 | mm-Gapdh | AGG TCG GTG TGA ACG GAT TTG | TGT AGA CCA TGT AGT TGA GGT CA |
| 5 | mm-Cyp7a1 | TGG GCA TCT CAA GCA AAC AC | TCA TTG CTT CAG GGC TCC TG |
| 6 | mm-Cyp27a1 | CCA GGC ACA GGA GAG TAC G | GGG CAA GTG CAG CAC ATA G |
| 7 | mm-Cyp7b1 | GAA AAC TCT TCA AAG GCA ACA TGG | ACT GGA AAG GGT TCA GAA CAA ATG |
| 8 | mm-Cyp8b1 | GAA GAT CCA CCA CTA CAG CAT | GGA CAA AGG TCT TCA TCT CG |
| 9 | mm-Hsd3b7 | GGG AGC TGC GTG TCT TTG A | GTG GAT GGT CTT TGG ACT GGC |
| 10 | mm-Slco2b1 | CTC AGG ACT CAC ATC AGG ATG C | CTC TTG AGG TAG CCA GAG ATC A |
| 11 | mm-Ch25h | TTA ACA TCT GGC TGT CGG TG | AGA GTG CCC AGC ATT TTG TC |
| 12 | mm-Shp | AAG GGC TTG CTG GAC AGT TA | TCT CTT CTT CCG CCC TAT CA |
| 13 | mm-Sirt1 | GCT GAC GAC TTC GAC GAC G | TCG GTC AAC AGG AGG TTG TCT |

**Supplementary Table 1** The primer sequences of the targeted genes.
